# Cellular therapies for sickle cell disease: Should we be calling them a cure?

**DOI:** 10.1002/hem3.70283

**Published:** 2025-12-08

**Authors:** Lydia O. Okoibhole, Funmi Dasaolu, Zainab Garba‐Sani, Stephen P. Hibbs

**Affiliations:** ^1^ Kavli Centre for Ethics, Science and the Public, Faculty of Education University of Cambridge Cambridge United Kingdom; ^2^ Wellcome Connecting Science Wellcome Sanger Institute Hixton United Kingdom; ^3^ Learning Disability & Patient Services Oxford University Hospitals NHS Foundation Trust Oxford United Kingdom; ^4^ Florence Nightingale Faculty of Nursing, Midwifery & Palliative Care King's College London London United Kingdom; ^5^ Stanford Medicine Stanford California United States; ^6^ Sickle Cell Society London United Kingdom; ^7^ Wolfson Institute of Population Health Queen Mary University of London London United Kingdom

Recent advances in gene therapy and expanded access for haematopoietic stem cell transplantation (HSCT) have made conversations about a *cure* more pertinent for individuals living with sickle cell disease (SCD). These advances, including the approval of exa‐cel gene therapy in the United Kingdom,^1^ have amplified the language of cure within policy, media, and clinical discussions. However, as this language becomes more visible, questions emerge about what *cure* means for individuals living with SCD, for healthcare systems, and for communities historically underserved and marginalized within research and clinical care.

The term *cure*, derived from the Latin “curare,” meaning “to care for” or “to heal,” suggests the complete elimination of symptoms caused by disease and a restoration to a previous state of health,^2^ but in the context of a lifelong disease like SCD, the meaning is more complex.

In this article, we begin with the personal reflections of one author (F.D.) living with SCD as she grapples with the possibility and uncertainty of cellular therapies (Box 1). Next, we survey how the language of cure is used in public discussions about SCD cellular therapies. We share the account of one author (Z.G.‐S.) whose experience befits the word *cure*, before exploring complexities that challenge the use of curative language. Finally, we highlight important considerations for health professionals and health systems relating to cellular therapies for SCD.

Box 1 **What might cure mean for me? (Funmi Dasaolu)**

Curative treatments…. two words that hold unimaginable, incomprehensible change *if successful*…. When I think about the word curative, I think about no more Sickle Cell, the blood disorder that has been so pivotal and formative, equally shaping and plaguing my life. I think about no more excruciating pain crises, hospitalizations, endless medical appointments, red cell exchange transfusions, and medications.

I dream of FREEDOM, freedom of choice and spontaneity, no longer being steered by my body's limitations. Most importantly, freedom from fatigue, pain, and the daily considerations and evaluations that are essential given the unpredictability of the condition. The risk assessments required for life's routine activities like socializing, exercising, work, or relationships. I think of a better quality of life.

I also feel FEAR…understanding the transplant and gene therapy process, namely the risks of conditioning, the uncertainty and unpredictability of recovery causes unrivaled anxiety intertwined with hope. Will it work, will I die, will I be pain‐free?

If successful, it will radically change my life…. whilst I imagine these changes will be largely positive and hugely welcomed, I also wonder who will I be without Sickle Cell? How do I live a life without this condition? I have no schema for this.

Herein lies the problem, curative implies eradication of disease and a return to a previous state of health. But, Sickle Cell is all I have ever known, what am I returning to? The symptoms of avascular necrosis may stabilize after treatment, but the damage caused by Sickle Cell remains. And do family and friends suddenly forget the trauma of supporting their loved ones in decades past?

## HOW IS CURATIVE LANGUAGE USED IN DISCUSSIONS OF SICKLE CELL CELLULAR THERAPIES?

The last decade has seen a significant increase in discussion about curative therapies in SCD. In 2023, when the US Food and Drug Administration (FDA) approved the gene editing therapy exa‐cel, it was described as “the first CRISPR cure for a genetic disease.”^3^, ^4^ Similarly, in 2025, the UK National Institute for Health and Care Excellence (NICE) described exa‐cel's approval as a “potential cure for some people with severe sickle cell disease.”^5^ This approval followed a long and complex journey of regulatory review and appeal,^6^ which finally resulted in the National Health Service (NHS) funding this one‐time therapy,^5^ described by NHS England as a source of hope.^1^

Additionally, conversation continues to build around allogeneic HSCT in SCD, as developments in conditioning regimens increase accessibility for a much wider group of people, including older recipients, those without a fully matched donor and those with additional comorbidities.^7^ In a patient guide addressing SCD and HSCT, UK charities Anthony Nolan and the Sickle Cell Society described the treatment as a cure, while also defining the benefits and risks associated.^8^ However, medical regulators, healthcare professionals, and media reports rarely explicitly define cure, and the potential for misunderstanding is significant.

A 2024 scoping review identified 64 scientific articles about SCD and beta thalassemia gene editing and HSCT‐based treatments that used curative language.^9^ Only a minority (11%) offered an explicit definition of “cure,” and their interpretations differed markedly, encompassing varying criteria^9^ such as:

– “no subsequent disease‐related expenditures”
– “replaces the defective product with normal cells”
– “functional graft” and “developed persistent mixed chimerism”
– “completely suppresses disease‐related complications and costs and restores life expectancy and health‐related quality of life to that of a comparable individual unaffected by the disease.”

Each of these definitions has a different focus: economic, biomedical, or holistic, raising the question—what does *cure* really mean in SCD, and is it an appropriate use of the term?

## WHY MIGHT CURE BE THE RIGHT WORD?

Cellular therapies can be **transformative**. For patients who have endured repeated hospital admissions for pain crises, transfusions every few weeks, or progressive organ complications, successful treatment can mean freedom from these burdens. Cellular therapies have been shown to dramatically reduce or even eliminate vaso‐occlusive crises, improve hemoglobin levels, and prevent further organ damage.^10^ Patients have described being able to return to education, work, and family life with a new sense of stability and independence.^11^ This transformation experienced by some individuals is reflected by the word *cure*. Furthermore, the language of cure can raise visibility and hope within a patient community who suffer chronic underinvestment in research, a dearth of therapies, and withdrawal of other novel treatment options. Below, one of the authors (Z.G.‐S.) shares why *cure* accurately describes her life after HSCT (Box 2).

Box 2 **The meaning of cure (Zainab Garba‐Sani)**

One month post stem cell transplant, I was still in survival mode—barely coping after already a month in hospital, including two harrowing weeks in ICU. I was tethered to half a dozen IV lines, reliant on intravenous nutrition, and utterly exhausted from the hardest thing I'd ever endured. Then, one morning on ward round, my doctor told me that only 4% of my hemoglobin was sickle. I sat there in shock. Suddenly, I remembered what all of this was for. I wasn't fighting for my life for nothing. I hadn't gone through eighteen months of complex red cell exchanges, three rounds of fertility preservation, and 11 days of chemotherapy, radiotherapy, and immunotherapy in vain. The long nights in agonizing pain, the days battling sepsis, the hours spent hemorrhaging blood—they all had purpose. In that moment, I saw it: a faint light at the end of a long, dark tunnel. This was the beginning of the rest of my life—a life *after* sickle cell.

Seven months post‐transplant, I went out for some fresh air. As I walked, my steps quickened. Before I realized it, my legs began to jog. Then, I was running. My heart didn't feel like it was about to burst; my lungs didn't ache; breathing didn't hurt. It was effortless—joyful even. In that moment I thought, maybe this new Zainab, the one discharged from sickle cell care—the *cured* Zainab—runs. And if she runs, what else might she do? It's moments like that when I'm reminded of the power of the word *cure*. It's not just medical; it's emotional. It carries hope, renewal, and the promise of a fuller, healthier future.

Eight months post‐transplant, after an ophthalmology review and MRI scan, I was told that my retinopathy and aneurysm were stable. Relief washed over me. For years, those complications had hung over me like a shadow—powerful reminders that at any moment, something could change, and I could lose parts of myself I cherished. The fear of what worsening complications could take from me—my sight, my mind, my independence, my quality of life—was one of the driving forces behind my decision to pursue a cure. To hear that they were no longer progressing lifted a weight I had carried for years. For the first time, I could imagine a future not dictated by constant fear that everything might collapse in an instant. A cure, for me, is not about erasing the past—it's about the invitation to finally slow down, to breathe, and to live freely in the present.

*Cure* is a strong word, and rightly so; it carries weight, history, and complexity. I understand why many approach it with caution—because cure can imply finality or suggest that suffering is easily undone. But for me, its strength lies precisely in its difficulty. The word *cure* reflects the magnitude of what it takes to reach that point—the uncertainty, the risk, the physical and emotional toll of pursuing something that is never promised. Where the stakes are this high, no cure is ever simple or guaranteed; not in cancer, not in sickle cell, not in any chronic condition I can think of. Some people relapse, some don't make it, and others find their lives changed in ways they never imagined. To me, *cure* captures both the possibility and the peril of transformation—the extraordinary intersection of science, endurance, and hope. The reality is that the enormity of the curative journey requires sacrifice. Individually to systemically, cures come at a cost but offer such promise. By including sickle cell in the curative discussion, we honor not only the scientific achievement and decades of advocacy, but also the human courage such a decision demands.

## WHY MIGHT CURE BE THE WRONG WORD?

Cellular therapies cannot fix all physical symptoms. For example, many people live with complications from SCD, such as nephropathy, hepatopathy, or cognitive impairment, which cellular therapies may stabilize but not reverse.^12^ Additionally, conditioning regimens involving chemotherapy, immunotherapy, and radiotherapy may create new physical impairment, such as infertility, secondary cancers, and other long‐term complications. If individuals receiving allogeneic HSCT develop chronic graft‐vs‐host disease, they exchange one severe chronic disease for another.^12^ Furthermore, additional harms may yet emerge—such as the reported increase in potential driver mutations linked to myeloid neoplasms following SCD gene therapy.^13^ These clinical limitations are reflected in lived experience.

The tension between curative language and lived reality is illustrated in the story of Genesis Jones, who underwent HSCT for SCD in the United States.^3^ Although her transplant successfully engrafted, Jones was subsequently diagnosed with secondary cancer. She continued to struggle with chronic pain and symptoms of organ damage previously caused by SCD. Furthermore, once she was no longer categorized as having SCD, her access to some forms of healthcare was removed.

Jones reported struggling with her identity and mental health after her treatment, feeling “in‐between” and no longer truly part of the SCD community.^3^ As people are born with SCD, it profoundly shapes identities, influences personal narratives, social interactions, and community dynamics from birth.^14^ Though often framed in terms of cure, SCD treatments require individuals to negotiate a new identity shaped by both the legacy of their condition and the transformations treatment may bring.

Existing relational dynamics with family members, friends, and carers may be disrupted and require significant adaptation.^15^, ^16^ As Jones's experience illustrates, once an individual is deemed to be “cured,” this may constrain the support they can access. For example, a person may have been eligible for disability financial support before treatment but no longer qualifies after. Yet the long‐term complications of SCD, including chronic pain, may still profoundly shape their daily life, highlighting how a biomedical cure may not fully align with lived reality or ongoing support needs.

## CHOOSING WORDS WITH CARE

Is *cure* the most appropriate term in the context of cellular therapies in SCD? For some individuals receiving these treatments, including one of the authors, the answer is yes—cure is a helpful and necessary word, which both reflects the gravity of such therapies and brings much‐needed hope and attention for the wider community. Yet for others, cure language can create false expectations, undermine the experiences of survivors, their extended community, and clinicians, and shut down important wider conversations.

Crucially, the conversation around cellular therapies in SCD is happening against a backdrop of historic inequalities, health inequity, and mistrust. Black communities—disproportionately affected by SCD—face systemic racism, neglect, and suspicion in research and healthcare.^17^, ^18^ This context shapes how curative language is interpreted and received, which heightens the need for careful and transparent communication. Addressing this context is essential to any discussion of cure language, not only in terms of ensuring equitable access to emerging therapies, but also in rebuilding relationships of trust and accountability between researchers, clinicians, and communities. Without recognizing and actively addressing these systemic injustices, the promise of curative treatments risks reproducing the same inequities that have historically shaped the experiences of those living with SCD.

Finally, in the absence of thoughtful implementation, the language of cure risks obfuscating—and even exacerbating—the deep unmet needs faced by many people living with SCD. These treatments remain inaccessible for the vast majority of individuals with SCD due to age, comorbidities, cost, or healthcare system infrastructure and expertise. This is especially true within low‐ and middle‐income countries, where most people living with SCD reside. Alongside the development of cellular therapies, investment in accessible treatment, management, and genetic counseling remains critical.

To conclude, despite the challenges with the word cure, there is no simple, perfect alternative. The terms “transformative therapies” or “radical therapies” may be less prone to causing unrealistic expectations. But perhaps the most important goal is to make visible the complexity, resilience, and dignity of living with SCD, whether someone receives cellular therapy or not. Individuals considering these treatments need transparent and carefully communicated information to make informed choices. Health systems need to consider how to holistically support patients undertaking cellular therapies from decision‐making through to survivorship. Beyond terminology, it is critical to create space for honest, nuanced conversations about motivations, expectations, needs, and life after treatment.

## AUTHOR CONTRIBUTIONS

**Lydia O. Okoibhole**: Conceptualization; writing—review and editing; writing—original draft. **Funmi Dasaolu**: Writing—review and editing; writing—original draft. **Zainab Garba‐Sani**: Writing—review and editing; writing—original draft. **Stephen P. Hibbs**: Conceptualization; writing—original draft; writing—review and editing.

## CONFLICT OF INTEREST STATEMENT

The authors report no conflict of interests.

## FUNDING

L.O.O. is supported by the Wellcome Trust (Grant number 220540/Z/20/A to the Wellcome Sanger Institute and Wellcome Connecting Science). F.D. is supported by an NIHR Pre‐Doctoral Clinical Academic Fellowship, Award Number NIHR304921. S.P.H. is supported by a HARP doctoral research fellowship, funded by the Wellcome Trust (Grant number 223500/Z/21/Z).

## Data Availability

Data sharing is not applicable to this article as no datasets were generated or analyzed during the current study.
